# Biochemical, anthropometric and body composition indicators as predictors of hepatic steatosis in obese adolescents

**DOI:** 10.1590/0103-0582201432215813

**Published:** 2014-06

**Authors:** Amanda Oliva Gobato, Ana Carolina J. Vasques, Roberto Massao Yamada, Mariana Porto Zambon, Antonio de Azevedo Barros-Filho, Gabriel Hessel

**Affiliations:** 1 Faculdade de Ciências Médicas da Unicamp, Campinas, SP, Brasil

**Keywords:** fatty liver, obesity, adolescent, enzymes

## Abstract

**OBJECTIVE:**

To describe the prevalence of hepatic steatosis and to assess the performance of biochemical, anthropometric and body composition indicators for hepatic steatosis in obese teenagers.

**METHODS:**

Cross-sectional study including 79 adolecents aged from ten to 18 years old. Hepatic steatosis was diagnosed by abdominal ultrasound in case of moderate or intense hepatorenal contrast and/or a difference in the histogram ≥7 on the right kidney cortex. The insulin resistance was determined by the Homeostasis Model Assessment-Insulin Resistance (HOMA-IR) index for values >3.16. Anthropometric and body composition indicators consisted of body mass index, body fat percentage, abdominal circumference and subcutaneous fat. Fasting glycemia and insulin, lipid profile and hepatic enzymes, such as aspartate aminotransferase, alanine aminotransferase, gamma-glutamyltransferase and alkaline phosphatase, were also evaluated. In order to assess the performance of these indicators in the diagnosis of hepatic steatosis in teenagers, a ROC curve analysis was applied.

**RESULTS:**

Hepatic steatosis was found in 20% of the patients and insulin resistance, in 29%. Gamma-glutamyltransferase and HOMA-IR were good indicators for predicting hepatic steatosis, with a cutoff of 1.06 times above the reference value for gamma-glutamyltransferase and 3.28 times for the HOMA-IR. The anthropometric indicators, the body fat percentage, the lipid profile, the glycemia and the aspartate aminotransferase did not present significant associations.

**CONCLUSIONS:**

Patients with high gamma-glutamyltransferase level and/or HOMA-IR should be submitted to abdominal ultrasound examination due to the increased chance of having hepatic steatosis.

## Abstract (es)

**OBJETIVO:**

Describir la prevalencia de la esteatosis hepática y evaluar el desempeño de indicadores bioquímicos, antropométricos y de composición corporal para identificar la enfermedad en adolescentes obesos.

**MÉTODOS:**

Estudio transversal con 79 adolescentes de 10 a 18 años. Se diagnosticó la esteatosis hepática por ultrasonido abdominal en caso de contraste hepatorrenal moderado o intenso y/o diferencia en el histograma ≥7 con relación al córtex del riñón derecho. Se determinó la resistencia a la insulina por el índice *Homeostasis Model Assessment-Insulin Resistence *(HOMA-IR) para valores >3,16. Los indicadores antropométricos y de composición corporal fueron índice de masa corporal, porcentaje de grasa corporal, circunferencia abdominal y grasa subcutánea. Se dosificaron glucemia e insulina en ayuno, perfil lipídico y las encimas hepáticas aspartato aminotransferasa, alanina aminotransferasa, gama-glutamiltransferasa y fosfatasa alcalina. Se analizaron curvas ROC para evaluar el desempeño de los indicadores en identificar adolescentes con esteatosis hepática.

**RESULTADOS:**

La esteatosis hepática estuvo presente en el 20% de los pacientes y la resistencia a la insulina, en el 29%. La gama-glutamiltransferasa y el HOMA-IR se mostraron buenos indicadores para predecir la esteatosis hepática, con punto de corte de 1,06 veces por encima del valor de referencia para la gama-glutamiltransferasa y de 3,28 para el HOMA-IR. Los indicadores antropométricos, el porcentaje de grasa corporal, el perfil lipídico, la glucemia y la aspartato aminotransferasa no presentaron diferencias significantes.

**CONCLUSIONES:**

Pacientes con elevación de gama-glutamiltransferasa y/o HOMA-IR deben ser sometidos al examen de ultrasonido abdominal con gran probabilidad de obtenerse como resultado la esteatosis hepática.

## Introduction

The non-alcoholic fatty liver disease (NAFLD) is a clinicopathological condition characterized by lipid accumulation inside the hepatocytes, whose spectrum of presentation ranges from a simple fatty infiltration of liver (steatosis), through the establishment of an inflammation (steatohepatisis), to fibrosis and hepatic cirrhosis. The pathologic condition is similar to that of the alcohol-induced lesion, but it occurs in individuals without significant alcohol consumption^(^ 1 ^)^.

NAFLD occurs mainly in obese individuals and it is an increasing problem in children and adolescents, given the increasing prevalence of childhood obesity. The hepatic steatosis (HS) with onset in childhood and adolescence deserves special attention because most patients are asymptomatic and the evolution is silent^(^ 2 ^)^.

The gold standard for the diagnosis of NAFLD is liver biopsy; however, due to the difficulty of implementation and the risk of complications, indirect methods such as imaging and laboratory exams associated to the history and clinical examination, have been widely used in children and adolescents^(^ 3 ^)^.

The difficulty to diagnose coupled with the small number of studies available makes the prevalence of HS still poorly known in the pediatric age group. HS seems to be primarily associated with obesity and insulin resistance (IR). With the significant increase in the prevalence of obesity in children and adolescents and the direct relationship of this nutritional disorder with the development of HS, the present study aimed to describe the prevalence of HS diagnosed by abdominal ultrasonography and to evaluate the performance of biochemical, anthropometric, and body composition indicators in identifying HS in obese adolescents.

## Method

We conducted a cross-sectional study with adolescents attending the Child and Adolescent Obesity Outpatient Clinic at Hospital de Clínicas from the School of Medical Sciences within Universidade Estadual de Campinas (Unicamp). All adolescents treated from April 2011 to May 2012 who presented body mass index (BMI) ≥P_97_ for age and sex, according to the charts of the Word Health Organization (WHO^(^ 4 ^)^, were invited to participate in the research. We analyzed the clinical, laboratory, and ultrasonography characteristics of 79 patients of both sexes, from 10 to 18 years old, who were diagnosed with obesity and whose parents signed an informed consent form (ICF). 

Anthropometric techniques for measuring weight and height were those recommended by Lohman et al^(^ 5 ^)^. We calculated BMI by the Quetelet index (BMI=weight/height^2)^. Waist circumference (WC) was measured in centimeters with tape measure (Sanny*(r)*), at the midpoint between the last rib and the superior border of the iliac crest. The percentage of body fat (%BF) was measured by dual energy X-ray absorptiometry (DXA), with Hologic^(r)^ appliance, Discovery QDR Series n. 1005-75. 

Insulin resistance was diagnosed by Homeostasis Model Assessment-Insulin Resistance (HOMA-IR), which is the product of fasting insulin (mUI/mL) and fasting plasma glucose (mmol/L) divided by 22.5. Insulin Resistance was defined when the values were above 3.16^(^ 6 ^)^.

We carried out the ultrasonography by a Toshiba device, Power Vision 6000, through sector transducers of 3.75MHz and 5MHz. All exams were performed by the same examiner, with the patient in supine position after fasting for 12 hours. The diagnosis of HS was considered in case of moderate or intense hepatorenal contrast and/or difference ≥7 on the histogram of the relationship right lobe/cortex of the right kidney^(^ 7 ^)^.

Subcutaneous fat (SF) was measured by ultrasonography, with a 7.5MHz linear transducer. The transducer was positioned in the middle at 1cm above the navel line, without exerting any pressure on the abdomen, in order not to underestimate the measurement. SF was measured as the distance (cm) between the skin and the outer face of the fascia of the rectus abdominis muscles^(^ 8 ^)^.

For laboratorial analysis of the biochemical exams, blood samples by venipuncture were collected in the morning after fasting for 12 hours. We analyzed fasting plasma glucose and lipid profile by enzymatic colorimetric; the measurement of aspartate aminotransferase (AST) and alanine aminotransferase (ALT) was performed by kinetic method ultraviolet (U.V.). For alkaline phosphatase (ALP) and gama-glutamil transferase (GGT), we used the colorimetric kinetic method, all with the Roche Diagnostics^(r)^ Laboratory Reagent Kit. For determination of basal insulin, we used the reagents kit from the Siemens Healthcare Diagnostics^(r)^ Laboratory by the method of chemiluminescence.

Furthermore, we calculated the fatty liver index (FLI), a simple predictor, which assesses the presence of HS in the general population, proposed by Bedogni et al^(^ 9 ^)^ and validated for the adult population.

FLI=(e^0,953*log(TG)+0,139*IMC+0,718*log(GGT)+0,053*WC-15,745)^/(1+e^0,953*log(TG)+0,139*IMC+0,718*log(GGT)+0,053*WC-15,745)^*100, being WC: waist circumference; GGT: gama-glutamil transferase; TG: triglycerides; log: logarithm; e: Euler. According to this index, it is considered HS when the value is ≥60.

We analyzed the data using the IBM Statistical Package for the Social Sciences (SPSS) version 20.0. The descriptive analysis of continuous variables included the calculation of means and their respective standard deviations; for categorical variables, their percentage values were calculated. We used the Kolmogorov-Smirnov to assess the normality of the distribution of studied variables. We used the Mann-Whitney test to compare two independent groups. The hypothesis of dependence between categorical variables was assessed using the chi-square or Fischer's exact test, according to the expected frequencies. We calculated the values for Odds Ratio (OR) and their respective confidence intervals of 95% (95%CI) to assess the strength of dependence between categorical variables.

The analysis of ROC curves (Receiver Operating Characteristic Curve) was used to assess the performance of the anthropometric and biochemical indicators in identifying adolescents with HS. Given the difference of values of the hepatic enzymes according to age and sex in the analyzed adolescents, the values of these enzymes were transformed in number of times the upper limit of normality (n^o^ x). That is, the result of the serum concentration of the hepatic enzyme was divided by the number of the reference value according to age and sex. We considered the reference value for each sex and age range and divided the value found by the reference value. We used as reference the values preconized by Roche Diagnostics^(^ 10 ^)^. The areas under the ROC curves were calculated as proposed by Hanley and McNeil. We used a confidence interval of 95%. The sensibility and specificity values were calculated for all variables in the analysis of the ROC curves with significant results. The cutoff point with the highest sum of sensitivity and specificity was chosen to optimize the relationship between these two parameters, reflecting greater accuracy in diagnosis.

The Kappa (k) test was used as a measure of agreement between the ultrasound (US) and the FLI. The k value reflects the degree of concordance between the methods. Values ​​of k equal to 1 indicate perfect agreement between both methods and values equal to zero ​​indicate no correlation between the tested methods.

The level of significance established as a basis for decision was lower than 5% (*p*<0.05) for all tests.

The study was approved by the Research Ethics Committee of Medical Sciences at Unicamp in December 2010, under protocol n. 872/2010. 

## Results

We evaluated 79 patients, being 39 (49.4%) female and 40 (50.6%) male, aged between 10 and 18 years (mean 12.8), who attended the Child and Adolescent Obesity Outpatient Clinic of Hospital de Clínicas da Unicamp.

The HS diagnosed by abdominal ultrasonography was present in 16 patients (20.3%). The mean ALT, GGT, and ALP were significantly higher in patients with HS, as well as the HOMA-IR index. Indicators of body composition and lipid profile showed no significantly different among the groups with and without HS (Table 1).

**Table 1:**
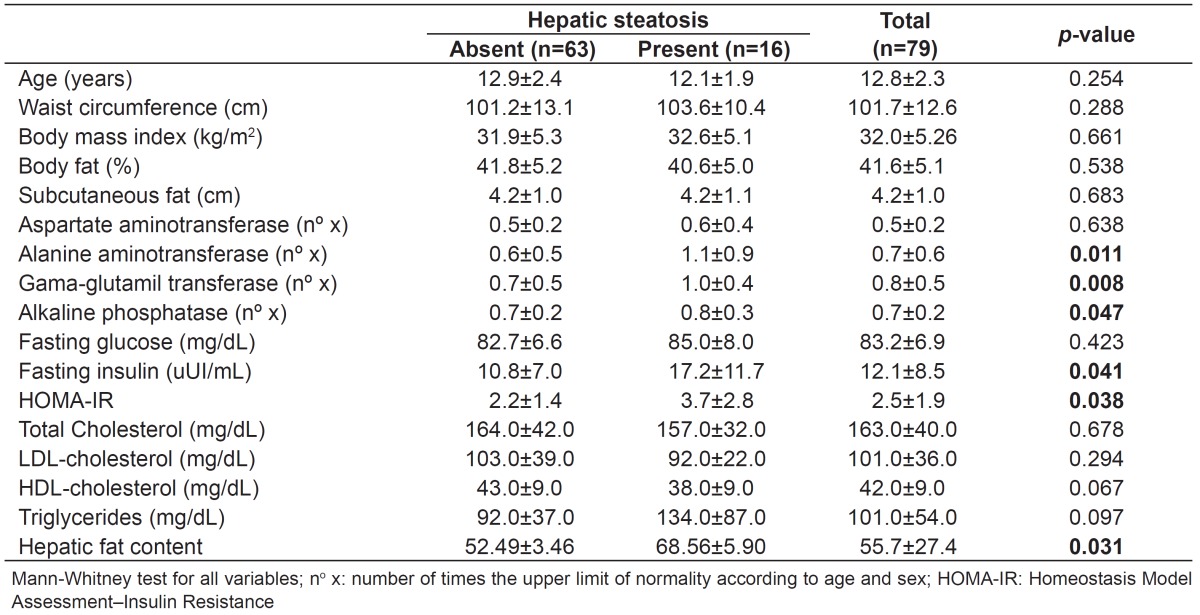
Clinical and laboratory characteristics of obese patients with and without hepatic steatosis. Values presented as means and standard deviation

Among the patients assessed, 23 (29.1%) presented IR, being 13 (56.5%) females and 10 (43.5%) males. Of the patients with diagnosis of IR, eight (34.8%) also had HS. The means for WC, BMI, SF and %BF were higher in patients who presented IR. Mean HDL-cholesterol was significantly lower in patients with IR (Table 2).

**Table 2:**
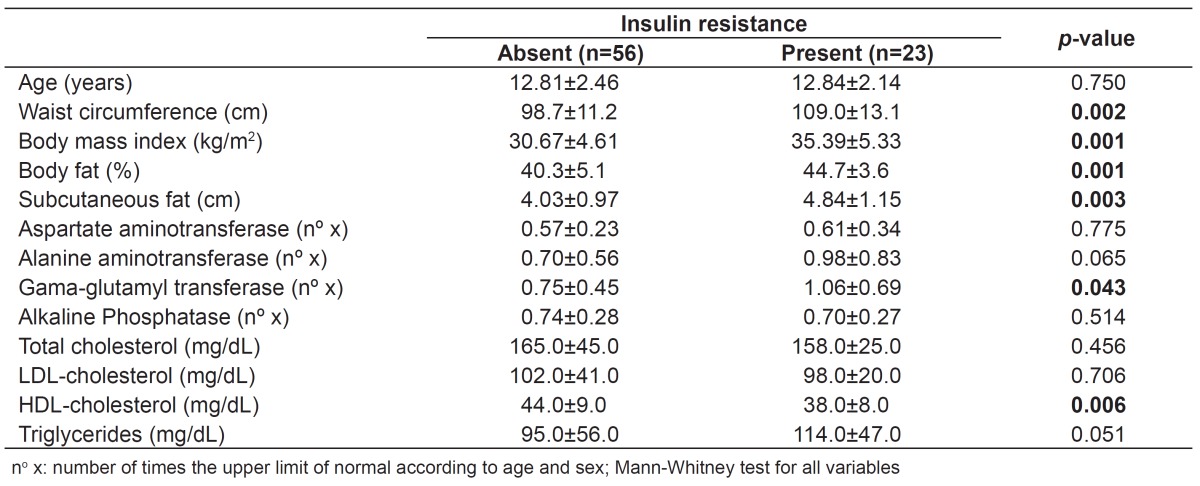
Characteristics of individuals evaluated according to insulin resistance

In the analysis of the ROC curve, liver enzymes (ALT, GG,T and ALP), HOMA -IR index, and FLI showed areas under the curve (AUC) significant (*p*<0.05) for the prediction of HS (Table 3). On the other hand, anthropometric and body composition indicators, plasma lipid profile, fasting glucose, and AST showed no significant results. The most accurate cutoff points for variables with significant results are shown in Table 3.

**Table 3:**
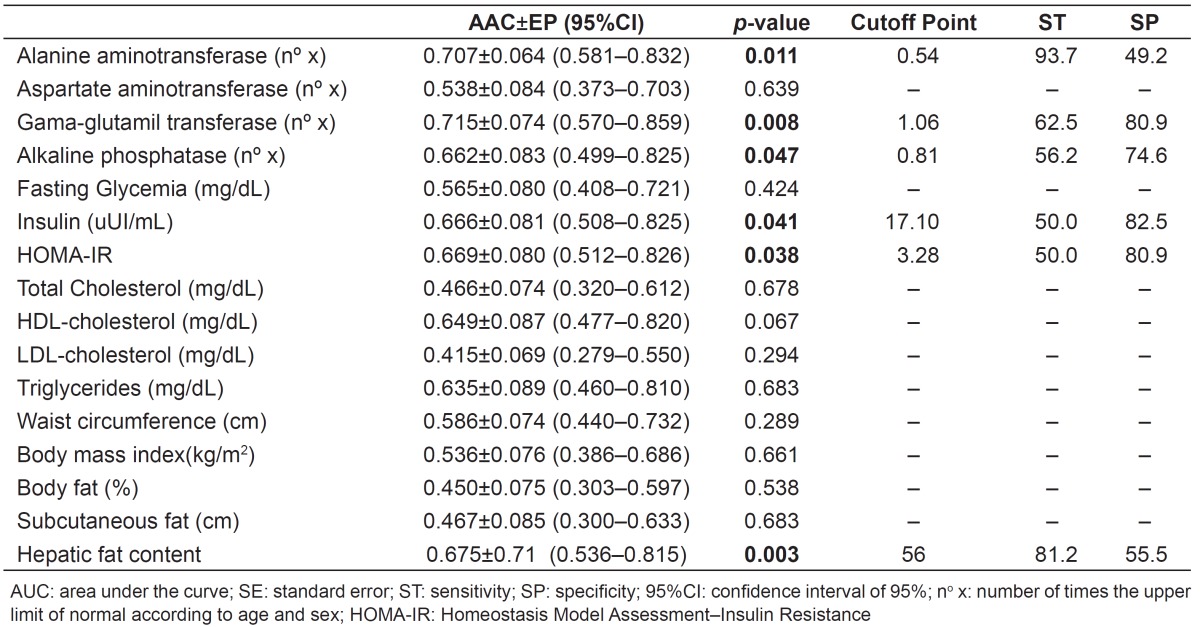
Effectiveness of biochemical, anthropometric, and body composition indicators and of the liver fat index in identifying hepatic steatosis

The concordance analysis between the diagnoses of HS by US and by FLI demonstrated a kappa index of 0.23, indicating little agreement between the methods. The chi-square test showed a correlation between both methods for the diagnosis of HS (6.924; *p*<0.009). Adolescents with a diagnosis of HS by FLI were five times more likely to show the HS by US. (OR 5.42; 95%CI 1.404-20.898).

## Discussion

The present study found a prevalence of HS of 20.3% in the studied population. The prevalence of HS is poorly understood and it may be related to the method used for diagnosis. The studies used elevated ALT levels as a criterion or the comparison of the echogenicity of the hepatic parenchyma with the renal cortex, the latter being more hyperechogenic in relation to the adjacent kidney. Studies show prevalence in obese children and adolescents from 15 to 42%^(^ 11 ^,^ 12 ^)^. The prevalence of NAFLD more than doubled in the past 20 years, rising from 3.9% in 1988 to 10.7% in 2010 in U.S. adolescents^(^ 13 ^)^. Based on autopsy studies, Schwimmer et al^(^ 14 ^)^ reported that the prevalence of HS in children and adolescents was of 9.6% and this prevalence rose to 38% in obese individuals. The difference in prevalence may be related to the degree of obesity, because in some studies with higher prevalence, mean BMI values are higher compared to studies with lower prevalence.

Abdominal ultrasonography is considered a good method for the diagnosis and monitoring of the degree of fatty infiltration in the liver, although it does not correlate with the degree of fibrosis. We adopted this method of image due to the good sensitivity (89%) and specificity (94%) for detection of HS compared to liver biopsy, considered as the gold standard^(^ 15 ^)^, besides having a relatively low cost, being noninvasive, easy to apply, and available in most services.

The prevalence of IR assessed by the HOMA-IR index, found in this study was 29.1%. Published data reported prevalence rates higher in obese adolescents^(^ 16 ^,^ 17 ^)^. This variation in the prevalence of IR in the studies may be explained by the lack of an established cutoff point as a reference to classify patient outcomes^(^ 6 ^-^ 18 ^)^.

HS seems to relate directly to IR. The mean for the HOMA-IR index in this study was significantly higher in patients with HS. The HOMA-IR was shown to be a good indicator in predicting HS, with a cutoff of 3.28, close to that used for diagnosing IR (HOMA-IR >3.16). There is increasing evidence that obesity and IR are risk factors for NAFLD also in children and adolescents. Seixas^(^ 19 ^)^ demonstrated that obese children and adolescents with SH are 2.6-fold more likely to present IR when compared with children without HS. El-Koofy et al^(20) ^observed that the prevalence of IR was significantly higher in patients with HS compared with patients without HS (73 versus 28%).

Insulin resistance and oxidative stress appear to be the two events involved in the pathogenesis of NAFLD. The pathophysiology involves two steps: first, IR causes steatosis; in the second, oxidative stress produces lipid peroxidation and active inflammatory cytokines, resulting in steatohepatitis^(^ 21 ^)^. The state of insulin resistance, often associated with obesity, leads to increased circulating free fatty acids that are seized and deposited within hepatocytes. This deposit activates the inflammatory cascade modulated by a variety of cytokines, and results in the exacerbation of oxidative stress, critical to the progression of NAFLD^(^ 3 ^)^. This hypothesis was confirmed in the study by Ruiz-Extremera et al^(^ 22 ^)^, in which it was observed in obese adolescents, that oxidative stress and IR are significant factors for the development of HS. Mager et al^(^ 23 ^)^ suggest that in all children and adolescents with overweight and obesity and that present IR, HS should be investigated.

In the comparison of means between groups with an without HS, the results of liver enzymes ALT, ALP, and GGT, presented as the number of times the upper limit of normal values, showed significant values. In the analysis of the ROC curve, the GGT showed greater specificity in the prediction of the HS, with a cutoff point 1.06 times higher than the reference value. ALT showed greater sensitivity, with a cutoff of 0.54 times - value below the reference value. As the cutoff is defined as the highest sum of sensitivity and specificity, when the cutoff of ALT rises to 1, the sensitivity decreases to 31.2%, indicating not being a good predictor of HS. The study by Ramos^(^ 24 ^)^ corroborates the findings in the present study. In his research, the mean values of GGT were also associated to HS, but no enzyme presented a good cutoff point to predict the HS. The author also highlights the importance of performing ultrasonography in children and adolescents as a criterion to evaluate HS.

Some studies have shown that changes in ALT and GGT can relate to some degree of liver inflammation, characterizing a more advanced stage of NAFLD^(^ 25 ^,^ 26 ^)^. Therefore, it is recommended that even in the absence of changes in serum levels of liver enzymes, the ultrasound integrate the overall assessment of the obese patient to identify mild cases of hepatic fatty infiltration.

The means of anthropometric indicators and body composition, and lipid profile showed no significant difference between groups, and neither did the ROC curve analysis. This can be explained by the fact that patients in this study were from a clinic that treats severer cases of obesity, all of which with BMI, WC, and %BF above the recommended, regardless of HS. El-Koofy et al^(^ 20 ^)^ observed a significant difference in the means of groups with and without HS for BMI, WC, and lipid profile. However, it is noteworthy that liver biopsy was the method used for the diagnosis of NAFLD and that, as inclusion criteria, all patients had to present hepatomegaly and/or abnormalities on ALT, indicating a more advanced stage of liver disease. The authors diagnosed 44% of patients with NAFLD, a much higher prevalence than that found in the present study. In contrast, the results of Duarte and Silva^(^ 12 ^)^ and Lin et al^(^ 27 ^)^ agree with the present study, with no significant difference in lipid profile between groups with and without HS.

The FLI showed little relation to the diagnosis of HS by US. This index uses three variables for the calculation (GGT, TG and WC), and two of these variables (TG and WC) showed no statistical difference between the groups with and without HS, which could be the explanation for the poor agreement between the methods. This is the first study that made reference to FLI in obese adolescents and due to the low concordance with the US, it was considered of little use in the studied population.

In conclusion, patients with elevated GGT and/or HOMA-IR >3.28 should undergo US examination with great probability of obtaining HS as a result. The FLI showed little association with the US, proving not to be a good method for the diagnosis of HS in obese adolescents.
