# Supplementary material for: Biochemical, anthropometric and body composition indicators as predictors of hepatic steatosis in obese adolescents
Source: Rev Paul Pediatr. 2014 Jun;32(2):230–6. doi: 10.1590/0103-0582201432215813 (PMC4183024; doi:10.1590/0103-0582201432215813)
Supplement: Translation

# Translation: Indicadores bioquímicos, antropométricos e de composição corporal como preditores da esteatose hepática em adolescentes obesos

**Authors:** Amanda Oliva Gobato, Ana Carolina J. Vasques, Roberto Massao Yamada, Mariana Porto Zambon, Antonio de Azevedo Barros-Filho, Gabriel Hessel

**Affiliations:** 1 Faculdade de Ciências Médicas da Unicamp, Campinas, SP, Brasil

**Keywords:** fígado gorduroso, obesidade, adolescente, enzimas

## Abstract

**OBJETIVO:**

Descrever a prevalência da esteatose hepática e avaliar o desempenho de indicadores bioquímicos, antropométricos e de composição corporal para identificar a doença em adolescentes obesos.

**MÉTODOS:**

Estudo transversal com 79 adolescentes de dez a 18 anos. Diagnosticou-se a esteatose hepática por ultrassom abdominal em caso de contraste hepatorrenal moderado ou intenso e/ou diferença no histograma ≥7 em relação ao córtex do rim direito. Determinou-se a resistência à insulina pelo índice *Homeostasis Model Assessment-Insulin Resistance* (HOMA-IR) para valores >3,16. Os indicadores antropométricos e de composição corporal foram: índice de massa corpórea, porcentagem de gordura corporal, circunferência abdominal e gordura subcutânea. Dosaram-se glicemia e insulina de jejum, perfil lipídico e enzimas hepáticas aspartato aminotransferase, alanina aminotransferase, gama-glutamiltransferase e fosfatase alcalina. Aplicou-se a curva ROC para avaliar o desempenho dos indicadores para identificar adolescentes com esteatose hepática.

**RESULTADOS:**

A esteatose hepática esteve presente em 20% dos pacientes e a resistência à insulina, em 29%. A gama-glutamiltransferase e o HOMA-IR mostraram-se bons indicadores para predizer a esteatose hepática, com ponto de corte de 1,06 vezes acima do valor de referência para a gama-glutamiltransferase e de 3,28 para o HOMA-IR. Os indicadores antropométricos, a porcentagem de gordura corporal, o perfil lipídico, a glicemia e a aspartato aminotransferase não apresentaram diferenças significantes.

**CONCLUSÕES:**

Pacientes com elevação de gama-glutamiltransferase e/ou HOMA-IR devem ser submetidos ao exame de ultrassom abdominal, havendo grande probabilidade de se obter como resultado a esteatose hepática.

## Introdução

A doença hepática gordurosa não alcoólica (DHGNA) é uma condição clínico-patológica caracterizada por acúmulo de lipídeos no interior dos hepatócitos, cujo espectro de apresentação varia desde uma simples infiltração hepática de gordura (esteatose), passando pelo estabelecimento de um processo inflamatório (esteato-hepatite) até fibrose e cirrose hepática. O quadro patológico lembra o da lesão induzida por álcool, mas ocorre em indivíduos sem ingestão etílica significativa^(^ ^1^ ^)^.

A DHGNA ocorre principalmente em indivíduos obesos e é um problema crescente em crianças e adolescentes, tendo em vista o aumento da prevalência da obesidade infantil. A esteatose hepática (EH) com início na infância e na adolescência merece atenção especial, pois a maioria dos pacientes é assintomática e a evolução, silenciosa^(^ ^2^ ^)^.

O padrão-ouro para o diagnóstico da DHGNA é a biópsia hepática; contudo, devido à dificuldade de execução e ao risco de complicações, métodos indiretos, como exames de imagem e laboratoriais, associados à história e exame clínico, têm sido amplamente utilizados em crianças e adolescentes^(^ ^3^ ^)^.

A dificuldade no diagnóstico aliada ao pequeno número de estudos disponíveis torna a prevalência da EH ainda pouco conhecida na faixa etária pediátrica. A EH parece estar principalmente associada à obesidade e à resistência à insulina (RI). Com o aumento expressivo da prevalência de obesidade em crianças e adolescentes e a relação direta desse distúrbio nutricional com o desenvolvimento da EH, o presente estudo teve como objetivo descrever a prevalência da EH diagnosticada por ultrassonografia abdominal e avaliar o desempenho de indicadores bioquímicos, antropométricos e de composição corporal para identificar a EH em adolescentes obesos.

## Método

Realizou-se estudo do tipo transversal com adolescentes atendidos no Ambulatório de Obesidade na Criança e no Adolescente do Hospital de Clínicas da Faculdade de Ciências Médicas da Universidade Estadual de Campinas (Unicamp). Todos os adolescentes atendidos de abril de 2011 a maio de 2012 que apresentaram índice de massa corpórea (IMC) ≥P_97_ para idade e sexo, segundo os gráficos da *Word Health Organization**^(^* ^4^ ^)^, foram convidados a participar da pesquisa. Analisaram-se características clínicas, laboratoriais e ultrassonográficas de 79 pacientes de ambos os sexos, de dez a 18 anos, com diagnóstico de obesidade e cujos responsáveis assinaram o termo de consentimento livre e esclarecido (TCLE). 

As técnicas antropométricas para aferir peso e altura foram as recomendadas por Lohman *et al**^(^* ^5^ ^)^. Calculou-se o IMC pelo índice de Quetelet (IMC=peso/estatura^2)^. Mediu-se a circunferência abdominal (CA) em centímetros com fita métrica (Sanny^(r))^, no ponto médio entre a última costela e a borda superior da crista ilíaca. A porcentagem de gordura corporal (%GC) foi mensurada pela absorciometria por dupla emissão de raios-X (DXA), com aparelho Hologic^(r)^, modelo Discovery QDR Series nº 1005-75.

Diagnosticou-se a RI pelo índice *Homeostasis Model Assessment-Insulin Resistance* (HOMA-IR), que é o produto da insulina de jejum (mUI/mL) e da glicemia de jejum (mmol/L) dividido por 22,5. A RI foi definida quando os valores se situavam acima de 3,16^(^ ^6^ ^)^.

Realizou-se o exame ultrassonográfico por meio de aparelho Toshiba, Power Vision 6000, através de transdutores setorial de 3,75MHz e linear de 5MHz. Todos os exames foram realizados pelo mesmo examinador, com o paciente em posição supina após jejum de 12 horas. O diagnóstico de EH foi considerado em caso de contraste hepatorrenal moderado ou intenso e/ou diferença ≥7 no histograma da relação lobo direito/córtex do rim direito^(^ ^7^ ^)^.

Mensurou-se a gordura subcutânea (GS) por ultrassonografia, com transdutor linear de 7,5MHz. O transdutor foi posicionado na linha média a 1cm acima da cicatriz umbilical, sem exercer nenhuma pressão sobre o abdome, a fim de não subestimar a aferição. Mediu-se a GS como a distância (cm) entre a pele e a face externa da fáscia dos músculos reto abdominais^(^ ^8^ ^)^.

Para análise laboratorial dos exames bioquímicos, coletaram-se as amostras de sangue por punção venosa periférica pela manhã após jejum de 12 horas. Analisaram-se a glicemia de jejum e o perfil lipídico por método enzimático colorimétrico; a mensuração da atividade da aspartato aminotransferase (AST) e da alanina aminotransferase (ALT) foi efetuada por método cinético ultravioleta (U.V.). Para a fosfatase alcalina (FALC) e a gama-glutamiltransferase (GGT), empregou-se o método cinético colorimétrico, todos com kit de reagentes do Laboratório Roche Diagnostics^(r)^. Para dosagem da insulina basal, utilizou-se kit de regentes do Laboratório Siemens Health Care Diagnostics^(r)^ pelo método de quimioluminescência.

Além disso, calculou-se o índice de gordura hepática (IGH), um preditor simples, que avalia a presença de EH na população em geral, proposto por Bedogni *et al**^(^* ^9^ ^)^ e validado para a população adulta.

IGH=(e^0,953*log(TG)+0,139*IMC+0,718*log(GGT)+0,053*CA-15,745)^/ (1+e^0,953*log(TG)+0,139*IMC+0,718*log(GGT)+0,053*CA-15,745)^*100, sendo CA: circunferência abdominal; GGT: gama-glutamiltransferase; TG: triglicérides; log: logaritmo; e: Euler. De acordo com esse índice, considera-se EH quando o valor é ≥60.

Analisaram-se os dados no *software* IBM *Statistical Package for the Social Sciences* (SPSS) versão 20.0. A análise descritiva das variáveis contínuas compreendeu o cálculo das médias e dos seus respectivos desvios padrão; já para as variáveis categóricas, calcularam-se os seus valores percentuais. Utilizou-se o teste de Kolmogorov-Smirnov para avaliar a normalidade da distribuição das variáveis estudadas. Utilizou-se o teste de Mann-Whitney para comparar dois grupos independentes. A hipótese de dependência entre as variáveis categóricas foi avaliada pelos testes do qui-quadrado ou exato de Fischer, conforme as frequências esperadas. Calcularam-se os valores de *Odds Ratio* (OR) e seus respectivos intervalos de confiança de 95% (IC95%) para avaliar a força da dependência entre as variáveis categóricas.

A análise de curvas ROC (*Receiver Operating Characteristic Curve*) foi utilizada para avaliar o desempenho dos indicadores antropométricos e bioquímicos em identificar adolescentes com EH. Tendo em vista as variações de valores das enzimas hepáticas conforme a idade e o sexo nos adolescentes analisados, estes foram transformados em número de vezes o limite superior de normalidade (nº x). Ou seja, dividiu-se o resultado da concentração sérica da enzima hepática pelo valor de referência, conforme a idade e o sexo. Considerou-se o valor de referência para cada sexo e faixa etária e dividiu-se o valor encontrado pelo valor de referência. Utilizaram-se como referência os valores preconizados pela Roche Diagnostics^(^ ^10^ ^)^. As áreas abaixo das curvas ROC foram calculadas, assim com o seu intervalo de confiança de 95%. Os valores de sensibilidade e especificidade foram calculados para todas as variáveis estudadas na análise da curva ROC com resultado significante. O ponto de corte com maior soma entre sensibilidade e especificidade foi escolhido por otimizar a relação entre esses dois parâmetros, refletindo maior acurácia no diagnóstico.

O teste Kappa (k) foi usado como medida de concordância entre o ultrassom (US) e o IGH. O valor de k reflete a intensidade da concordância entre os métodos. Valores de k iguais a 1 indicam concordância perfeita entre ambos os métodos e valores iguais a zero indicam ausência de concordância entre os métodos testados.

O nível de significância adotado como base para decisão foi inferior a 5% (*p*<0,05) para todos os testes.

O estudo foi aprovado pelo Comitê de Ética em Pesquisa da Faculdade de Ciências Médicas da Unicamp em dezembro de 2010.

## Resultados

Avaliaram-se 79 pacientes, sendo 39 (49,4%) do sexo feminino e 40 (50,6%) do sexo masculino, com idades entre dez e 18 anos (média de 12,8), procedentes do Ambulatório de Obesidade na Criança e no Adolescente do Hospital de Clínicas da Unicamp.

A EH, diagnosticada por ultrassonografia abdominal, esteve presente em 16 pacientes (20,3%). As médias de ALT, GGT e FALC foram significantemente maiores nos pacientes com EH, assim como o índice HOMA-IR. Os indicadores de composição corporal e perfil lipídico não apresentaram médias significativamente diferentes entre os grupos com e sem EH (Tabela 1).

**Tabela 1:** Características clínicas e laboratoriais dos pacientes obesos com e sem esteatose hepática. Valores apresentados em médias e desvio padrão

Dos pacientes avaliados, 23 (29,1%) apresentaram RI, sendo 13 (56,5%) no sexo feminino e dez (43,5%) no sexo masculino. Dos pacientes com diagnóstico de RI, oito (34,8%) cursavam com EH. As médias de CA, IMC, GS e %GC foram maiores para os pacientes que apresentaram RI. A média do HDL-colesterol foi significativamente mais baixa nos pacientes com RI (Tabela 2).

**Tabela 2:** Caracterização dos indivíduos avaliados segundo a resistência à insulina

Na análise da curva ROC, as enzimas hepáticas (ALT, GGT e FALC), o índice HOMA-IR e o IGH apresentaram áreas abaixo da curva (AAC) significantes (*p*<0,05) para a predição da EH (Tabela 3). Por outro lado, os indicadores antropométricos e de composição corporal, o perfil lipídico plasmático, a glicemia de jejum e a AST não apresentaram resultados significantes. Os pontos de corte mais acurados para as variáveis com resultado significante encontram-se na Tabela 3.

**Tabela 3:** Eficácia dos indicadores bioquímicos, antropométricos e de composição corporal e do índice de gordura hepática em identificar a esteatose hepática

A análise de concordância entre os diagnósticos de EH pelo US e pelo IGH demonstrou índice kappa de 0,23, indicando pequena concordância entre os métodos. O teste do qui-quadrado demonstrou associação entre ambos os métodos para o diagnóstico de EH (*p*<0,009). Os adolescentes com o diagnóstico de EH pelo IGH apresentaram cinco vezes mais chance de apresentar EH pelo US (OR 5,42; IC95% 1,404-20,898).

## Discussão

A presente pesquisa encontrou prevalência de EH de 20,3% na população estudada. A prevalência da EH não é bem conhecida e pode se relacionar ao método utilizado para o diagnóstico. Os estudos usam como critério níveis elevados de ALT ou a comparação da ecogenicidade do parênquima hepático com o córtex renal, estando este mais hiperecogênico em relação ao rim adjacente. Pesquisam mostram prevalência em crianças e adolescentes obesos de 15 a 42%^(^ ^11^ ^,^ ^12^ ^)^. A prevalência de DHGNA mais que duplicou nos últimos 20 anos, subindo de 3,9% em 1988 para 10,7% em 2010, em adolescentes americanos^(^ ^13^ ^)^. Com base em estudos de necropsia, Schwimmer *et al**^(^* ^14^ ^)^ relataram que a prevalência de EH em crianças e adolescentes foi de 9,6% e tal prevalência subiu para 38% em obesos. A divergência na prevalência pode se relacionar ao grau de obesidade, pois, em alguns estudos com prevalências maiores, a média de valores do IMC mostra-se mais elevada em relação aos estudos com prevalências menores.

A ultrassonografia abdominal é considerada um bom método para o diagnóstico e o seguimento do grau de infiltração gordurosa no fígado, embora não se correlacione com o grau de fibrose. Adotou-se esse método de imagem devido à boa sensibilidade (89%) e especificidade (94%) para detecção de EH comparado à biópsia hepática, considerada como padrão-ouro^(^ ^15^ ^)^, além de apresentar custo relativamente baixo, ser não invasivo, de fácil aplicabilidade e disponível na maioria dos serviços.

A prevalência de RI, avaliada pelo índice HOMA-IR, encontrada neste estudo foi de 29,1%. Dados publicados na literatura relatam taxas de prevalência maiores em adolescentes obesos^(^ ^16^ ^,^ ^17^ ^)^. Essa variação na prevalência de RI nos estudos pode ser explicada pela falta de um ponto de corte estabelecido como referência para classificar os resultados dos pacientes^(^ ^6^ ^-^ ^18^ ^)^.

A EH parece se relacionar diretamente à RI. A média do índice HOMA-IR encontrado neste estudo foi significativamente maior nos pacientes com EH. O HOMA-IR mostrou ser um bom indicador na predição da EH, com um ponto de corte de 3,28, valor próximo do utilizado para diagnosticar a RI (HOMA-IR>3,16). Existem evidências crescentes de que a obesidade e a RI são fatores de risco para DHGNA também em crianças e adolescentes. Seixas^(^ ^19^ ^)^ demonstrou que crianças e adolescentes obesos com EH apresentam 2,6 vezes mais chances de apresentar RI quando comparadas às crianças sem EH. El-Koofy *et al**^(20) ^*observaram prevalência de RI significativamente maior em pacientes com EH, em comparação com aqueles sem EH (73 *versus* 28%).

A RI e o estresse oxidativo parecem ser os dois eventos envolvidos na patogênese da DHGNA. A fisiopatologia envolve dois passos: no primeiro, a RI provoca a esteatose; no segundo, o estresse oxidativo produz a peroxidação lipídica e citocinas inflamatórias ativas, resultando em esteato-hepatite^(^ ^21^ ^)^. O estado de resistência insulínica, frequentemente associado à obesidade, leva ao aumento dos ácidos graxos livres circulantes que são captados e depositados dentro do hepatócito. Esse depósito ativa a cascata inflamatória, modulada por uma série de citocinas, e resulta na exacerbação do estresse oxidativo, processo fundamental para a progressão da DHGNA^(^ ^3^ ^)^. Tal hipótese foi confirmada no estudo de Ruiz-Extremera *et al**^(^* ^22^ ^)^, no qual se observou, em adolescentes obesos, que o estresse oxidativo e a RI são fatores significativos para o desenvolvimento da EH. Mager *et al**^(^* ^23^ ^)^ sugerem que, em todas as crianças e adolescentes com sobrepeso e obesidade e que apresentam RI, deve-se investigar a EH.

Na comparação das médias entre os grupos com e sem EH, os resultados das enzimas hepáticas ALT, FALC e GGT, apresentados como o número de vezes o limite superior de normalidade, mostraram resultados significativos. Na análise da curva ROC, a GGT mostrou maior especificidade na predição da EH, com ponto de corte 1,06 vezes acima do valor de referência. Já a ALT mostrou maior sensibilidade, com ponto de corte de 0,54 vezes - valor abaixo da referência. Como o ponto de corte é definido como a maior soma entre a sensibilidade e a especificidade, quando o ponto de corte de ALT sobe para 1, a sensibilidade diminui para 31,2%, indicando não ser um bom preditor da EH. Os resultados obtidos por Ramos^(^ ^24^ ^)^ corroboram os dados encontrados no presente estudo. Em sua pesquisa, os valores médios de GGT também se associaram à EH, porém nenhuma das enzimas apresentou um bom ponto de corte para predizer a EH. O autor ainda destaca a importância de se realizar a ultrassonografia em crianças e adolescentes como critério para avaliar a EH.

Alguns trabalhos demonstram que alterações da ALT e da GGT podem se relacionar a algum grau de inflamação hepática, caracterizando um estágio mais avançado da DHGNA^(^ ^25^ ^,^ ^26^ ^)^. Por isso, recomenda-se que, mesmo em ausência de alteração dos níveis séricos das enzimas hepáticas, a ultrassonografia integre a avaliação global do paciente obeso para se identificarem casos leves de infiltração gordurosa hepática.

As médias dos indicadores antropométricos e de composição corporal e o perfil lipídico não apresentaram diferença significativa entre os grupos, assim como a análise da curva ROC. Isso pode ser explicado pelo fato de os pacientes deste estudo serem provenientes de um ambulatório que atende aos casos mais graves de obesidade, na qual todos possuem IMC, CA e %GC acima do recomendado, independentemente da EH. El-Koofy *et al**^(^* ^20^ ^)^ observaram diferença significativa nas médias dos grupo com e sem EH para IMC, CA e perfil lipídico. Mas vale ressaltar que a biópsia hepática foi o método utilizado para o diagnóstico da DHGNA e que, como critério de inclusão, todos os pacientes precisavam apresentar hepatomegalia e/ou ALT alterado, indicando um estágio mais avançado da doença hepática. Os autores diagnosticaram 44% dos pacientes com DHGNA, prevalência bem maior do que a encontrada no presente estudo. Em contrapartida, os resultados de Duarte e Silva^(^ ^12^ ^)^ e Lin *et al**^(^* ^27^ ^)^ concordam com os do presente trabalho, não havendo diferença significativa do perfil lipídico entre os grupos com e sem EH. 

O IGH apresentou pouca relação com o diagnóstico de EH pelo US. Esse índice utiliza três variáveis para o cálculo (GGT, TG e CA), sendo que duas dessas variáveis (TG e CA) não apresentaram diferença estatística entre os grupos com e sem EH, podendo ser essa a explicação para a baixa concordância entre os dois métodos. Este é o primeiro estudo que fez referência ao IGH em adolescentes obesos e, pela baixa concordância encontrada com o US, foi considerado de pouca utilidade na população estudada.

Em conclusão, pacientes com elevação de GGT e/ou com HOMA-IR >3,28 devem ser submetidos ao exame de US com grande probabilidade de se obter, como resultado, a EH. O IGH apresentou pouca relação com o US, não sendo um bom método para o diagnóstico de EH em adolescentes obesos.
